# Cu-Hemin Nanosheets and Indocyanine Green Co-Loaded Hydrogel for Photothermal Therapy and Amplified Photodynamic Therapy

**DOI:** 10.3389/fonc.2022.918416

**Published:** 2022-06-30

**Authors:** Shu Zhu, Shuntao Wang, Chunping Liu, Meng Lyu, Qinqin Huang

**Affiliations:** ^1^ Department of Molecular Pathology, The Second Affiliated Hospital of Zhengzhou University, Zhengzhou, China; ^2^ Department of Medical Ultrasound, Tongji Hospital, Tongji Medical College, Huazhong University of Science and Technology, Wuhan, China; ^3^ Department of Breast and Thyroid Surgery, Union Hospital, Tongji Medical College, Huazhong University of Science and Technology, Wuhan, China; ^4^ Department of Radiation and Medical Oncology, Hubei Key Laboratory of Tumor Biological Behaviors, Hubei Cancer Clinical Study Center, Zhongnan Hospital of Wuhan University, Wuhan, China

**Keywords:** indocyanine green, glutathione, photothermal therapy, photodynamic therapy, hydrogel

## Abstract

Near-infrared (NIR) organic small molecule indocyanine green (ICG) could respond well to 808 nm laser to promote local high temperature and ROS generation for realizing photothermal therapy (PTT)/photodynamic therapy (PDT). However, the high content of GSH in the tumor microenvironment (TME) limited the further therapeutic performance of ICG. Herein, injectable agarose *in situ* forming NIR-responsive hydrogels (CIH) incorporating Cu-Hemin and ICG were prepared for the first time. When CIH system was located to the tumor tissue through local injection, the ICG in the hydrogel could efficiently convert the light energy emitted by the 808 nm laser into thermal energy, resulting in the heating and softening of the hydrogel matrix, which releases the Cu-Hemin. Then, the over-expressed GSH in the TME could also down-regulated by Cu-Hemin, which amplified ICG-mediated PDT. *In vivo* experiments validated that ICG-based PDT/PTT and Cu-Hemin-mediated glutathione depletion could eliminate cancer tissues with admirable safety. This hydrogel-based GSH-depletion strategy is instructive to improve the objective response rate of PDT.

## Introduction

At present, the clinical treatment based on cancer has fallen into a great predicament, which has become a treatment difficulty and research hotspot in the current medical field. Tumor microenvironment (TME) is different from healthy tissue environment. For example, TME is usually weakly acidic with high content of GSH and high heat sensitivity, which has encouraged researchers to develop new treatments to treat tumors (1). In recent years, the emergence and rapid development of photothermal therapy (PTT) has provided new ideas for the treatment of solid tumors (2–4). PTT is a new photophysical technology that converts the light energy into heat energy through photothermal conversion reagent, and then generates a local high temperature at a specific lesion, thereby using the heat energy to directly kill tumor cells (5, 6). PTT is less invasive and more precise than traditional chemotherapy, surgery and radiotherapy (7–9). Many copper-based compounds have good photothermal efficiency and are used as PTAs, and molybdenum-based compounds are also widely used in PTT (10, 11). Among them, the photothermal reagent indocyanine green (ICG) has attracted the attention of researchers due to its excellent biocompatibility and photothermal properties as it has strong near-infrared (NIR) light absorption at 808 nm (12). Not only that, ICG can act as a photosensitizer and transfer the energy to the surrounding oxygen molecules to generate chemically active reactive oxygen species (ROS) (13, 14). ROS could interact with biofilms, destroy cell structure and function, and inactivate cellular proteins, thereby killing cancer cells (15–17). ROS (such as superoxide anion, hydroxyl radical, hydrogen peroxide) could induce apoptosis and even leads to cell necrosis through oxidative stress (18, 19). However, the high content of the reducing substance glutathione (GSH) in the tumor microenvironment (TME) neutralizes the toxicity of ROS and largely inhibits the efficiency of ICG-based PDT (17, 20, 21).

As a typical tumor marker, GSH is widely present in TME, it is the most abundant biological thiol in cells, and plays a crucial role in pathological processes and biochemical pathways (22, 23). It exists in both reduced and oxidized states, and more than 90% of cellular GSH exists in reduced form (24, 25). Therefore, the regulation of reduced glutathione (GSH) is the focus in tumor therapy. The intracellular concentration of GSH (≈10 mM in TME) in cancer cells is much higher than that in normal cells, therefore the effect of ROS-based therapeutics is easily regulated by GSH (24, 26). Nanomaterials can treat various diseases due to their particularity (18, 19, 27), to this end, researchers have developed various GSH-consumable nanomaterials to address this challenge. For example, Fan et al. designed a FeS_2_-based nanozyme with high glutathione oxidase (GSH-OXD) activity and enhanced its tumor permeability through folic acid (FA) modification, thereby disrupting the redox homeostasis of the tumor site (28). Thus, FeS_2_ effectively depletes intracellular GSH, and at the same time produces a large amount of **·**OH to kill tumors. Cai et al. developed novel Cu-Hemin-PEG-LA nanosheets to achieve synchronized GSH depletion and ferroptosis (29). As a new type of nanomaterial, Cu-Hemin has a good tumor killing effect, and the synthesis method is simple and convenient. It can kill tumor tissue well at low doses. Cu-Hemin depletes intracellular GSH and enhances oxidative stress levels. Therefore, Cu-Hemin could well cooperate with ICG to optimize the therapeutic window of individualized medicine, achieving powerful tumor ablation effect. However, it is difficult to co-deliver ICG and Cu-Hemin to tumor sites simultaneously.

Although the traditional intravenous injection of nanomaterials to the tumor site improves the therapeutic efficiency to a certain extent, the uncontrollable release of nanomaterials and the complex physiological environment of the body make the accumulation of nanocarriers in tumor tissue still less than 10% (30–32). Macroscopic drug delivery systems such as light-responsive hydrogels (LRH) are promising smart controlled release systems (33–35). The hydrogel-carrying nanomaterials can aggregate at the tumor site and achieve almost 100% drug penetration (31). And LRH could achieve controlled release carriers for precise on-demand treatment. For example, Zhu et al. simultaneously encapsulated the photothermal agent Pd-C SAzyme and the chemotherapeutic drug camptothecin (CPT) into agarose hydrogels to form a light-controlled oxidative stress amplifier, achieving better photothermal/immunotherapy (15). Notably, this stimuli-responsive hydrogel can tune the rate and extent of carrier release by changing external parameters such as laser power, irradiation time, gel size. Therefore, the simultaneous delivery of ICG and Cu-Hemin into tumor sites using hydrogels is an ideal macroscopic transport mode.

**Scheme 1:**
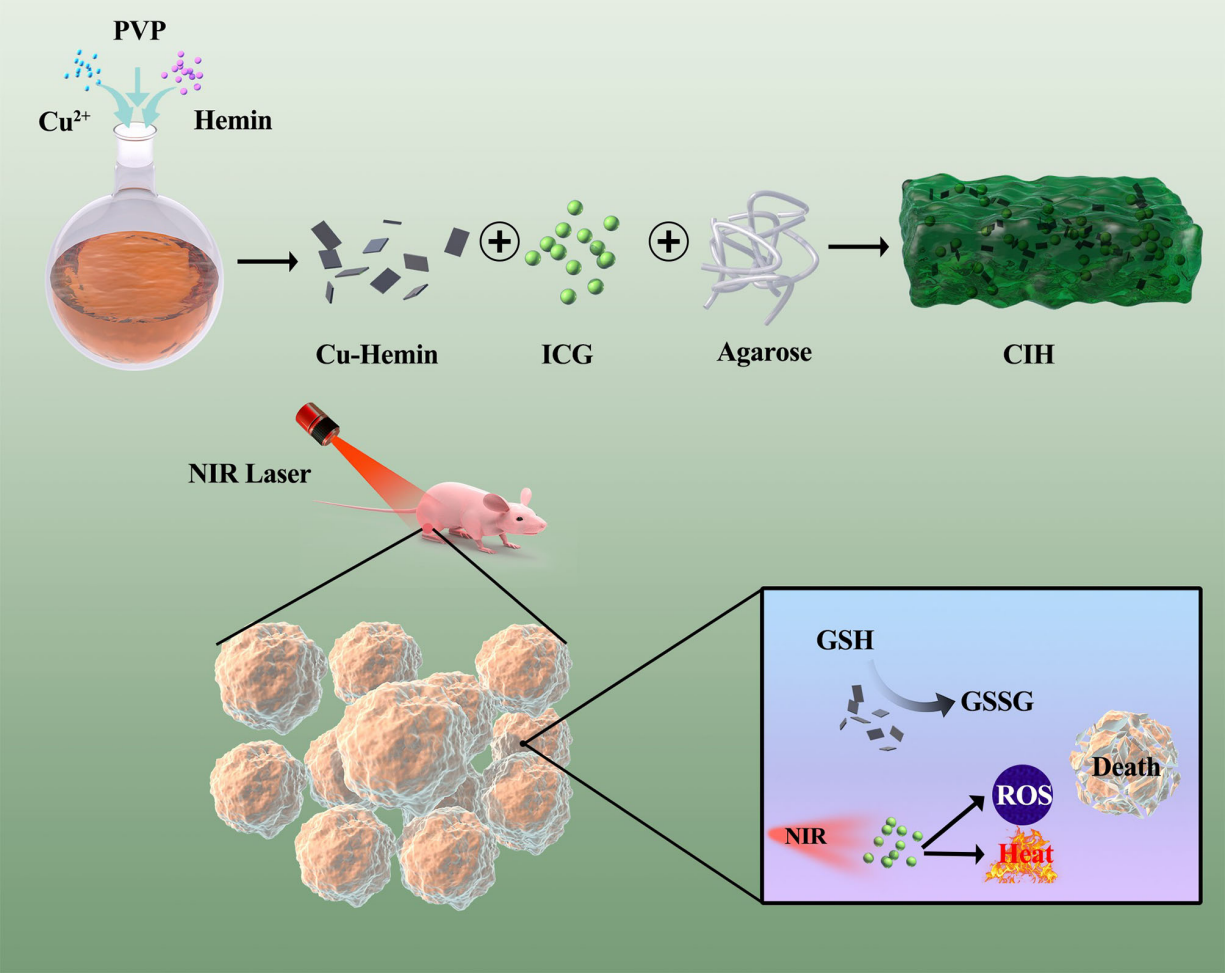
Schematic illustration of Cu-Hemin nanosheets and indocyanine green co-loaded hydrogel for photothermal therapy and amplified photodynamic therapy.

In this study, we performed a simple treatment of the tumor microenvironment regulator Cu-Hemin nanosheets and the photothermal agent ICG with agarose to form a composite hydrogel Cu-Hemin-ICG-hydrogel (CIH) (**Scheme 1**). CIH can stay at the tumor site for a long time after injection, and respond intelligently to 808nm laser. The ICG converts light energy into heat under NIR laser irradiation, and softens the hydrogel to release the Cu-Hemin while generating local high temperature in the mean time. ICG can also absorb 808 nm laser energy to stimulate surrounding oxygen molecules to generate ROS, and Cu-Hemin can amplify ROS generation by depleting intracellular GSH. This combination of modulating redox homeostasis and phototherapy achieved good tumor-suppressive effects in both *in vitro* and *in vivo* experiments. Importantly, agarose is regardas a safe drug approved by American Food and Drug Administration (FDA) (31). The experimental group of CIH combined with 808 nm laser did not have any adverse reactions during the treatment. This indicates that our designed therapeutic system achieves the unification of treatment and system safety. This way of ICG-based enhancing photothermal/photodynamic therapy by depleting GSH is instructive for the subsequent development of novel nano-therapeutic systems.

## Results and Discussion

**Figure 1:**
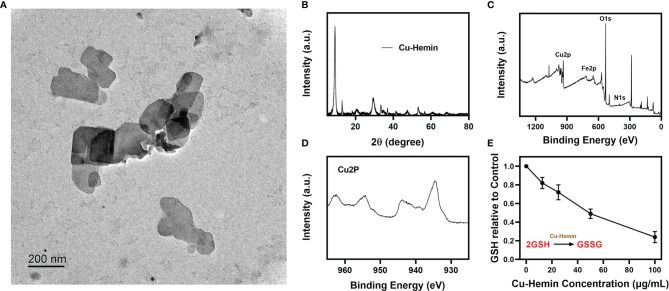
Characterization analysis of Cu-Hemin. **(A)** TEM image of Cu-Hemin nanosheets. **(B)** XRD pattern of Cu-Hemin. **(C)** The XPS spectrum of Cu-Hemin. **(D)** The Cu 2p XPS spectra of Cu-Hemin nanosheets. **(E)** The relative GSH content of the supernatant after the reaction of various concentrations Cu-hemin and 10 mm GSH in mixed solution for 24 h.

Cupric nitrate, PVP and hemin were mixed and stirred overnight, and the Cu-Hemin nanosheets were obtained by centrifugation (29). The transmission electron microscopy (TEM) images of Cu-Hemin are shown in **Figure 1A**, Cu-Hemin exhibits a random sheet-like structure with small nanoscale dimensions. The Cu-Hemin was characterized by XRD and XPS (**Figures 1B–D**), and the results showed that the Cu-Hemin was successfully prepared, which was similar to the Cu-Hemin Metal-organic frame (MOF) prepared by the predecessors (29). The results of zeta potential values (**Figure S1**) for Cu-Hemin nanosheets showed that our parpared Cu-MOF is stable. GSH, as an important intracellular regulatory metabolite, is overexpressed in tumor cells (36, 37). We configured different concentrations of Cu-Hemin to explore its ability to destroy GSH. As shown in the **Figure 1E**, GSH was depleted to different degrees after co-incubating with Cu-Hemin for several hours, and showed a concentration-dependent relationship. This result is encouraging that Cu-Hemin can respond to high intracellular levels of GSH and disrupt redox homeostasis after reaching the tumor site.

**Figure 2:**
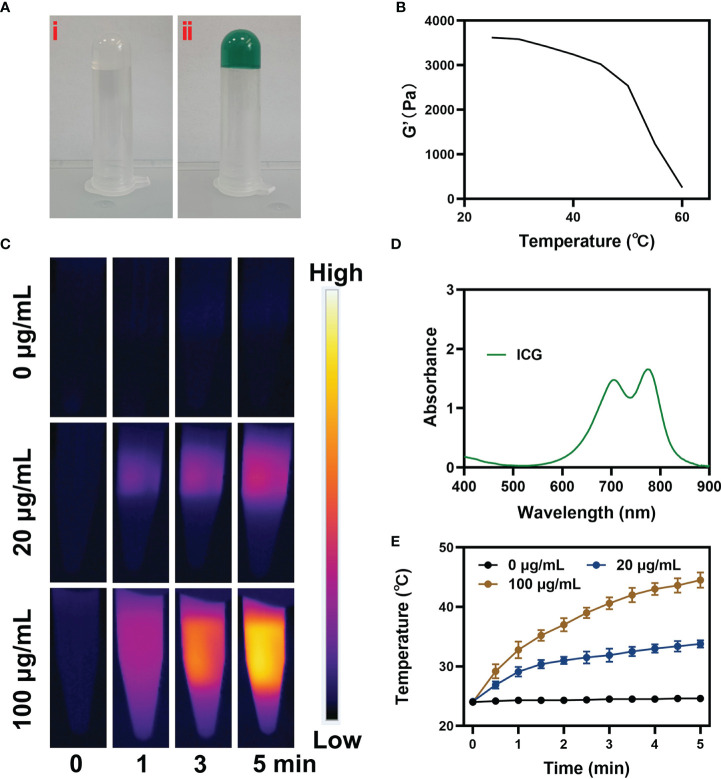
Characterization analysis of ICG. **(A)** The prepared image of pure hydrogel (i) and CIH (ii). **(B)** Rheological and temperature curves for the prepared CIH. **(C)** The corresponding thermal images and **(E)** photothermal heating curves of CIH at different concentrations (ICG: 0, 20 and 100 μg/mL) under an 808 nm (NIR-I) laser irradiation at a power density of 0.5 W/cm. **(D)** Absorption spectrum of ICG solution.

Then we prepared pure hydrogel and CIH by a simple method. As shown in the **Figure 2A**, the hydrogel will solidify at room temperature and will not flow down the tube wall, while the solidification performance of CIH with ICG and Cu-Hemin showed no difference. Scanning electron microscopy (SEM) results showed that the hydrogels displayed a typical porous structure with uniform distribution and neat arrangement of pore structures, which are essential in drug delivery and facilitate drug diffusion (**Figure S2**). Simultaneously, the storage modulus of this thermosensitive hydrogel varies with temperature. **Figure 2B** showed that the storage modulus of CIH decreases significantly at hyperthermia, which reflected that CIH softens with increasing temperature. We continued to study the time-temperature profiles of ICG solutions exposed to low-power NIR laser irradiation. Firstly, the absorption spectrum of ICG confirms that it has good absorption ability at 808 nm (**Figure 2D**). We configured different concentrations of ICG solutions (0, 20 and 100 μg/mL) and continuously exposed them to laser irradiation. Infrared thermal imaging (**Figure 2C**) shows that ICG can exert strong photothermal conversion performance under the irradiation of low-power laser. The temperature of the ICG solution (200 ug/mL) increased about 19°C (**Figure 2E**) after irradiation, thus confirming that ICG is an efficient photothermal conversion agent.

**Figure 3:**
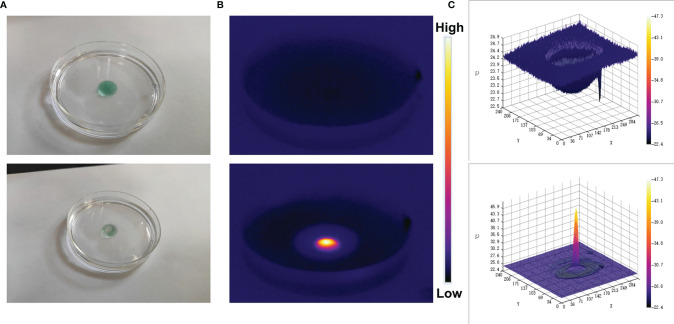
Digital image analysis of softening effect of CIH. **(A)** The morphology of the prepared CIH before and after 0.5 W/cm^2^ 808 nm laser irradiation for 5 min. **(B)** The infrared thermal images of the prepared CIH before and following irradiation. **(C)** Relevant 3D temperature diagram in 2B.

The good photothermal properties of ICG prompted us to move forward. We placed CIH in a glass dish, and after standing for a while, laser irradiation was performed, and infrared thermal images were recorded. As shown in the **Figures 3A, B**, the hydrogel temperature increased significantly after irradiation and gradually softened. Relevant 3D temperature diagram (**Figure 3C**) also verified similar results. This indicates that the encapsulated ICG has satisfactory photothermal performance and can be used for subsequent investigations.

**Figure 4:**
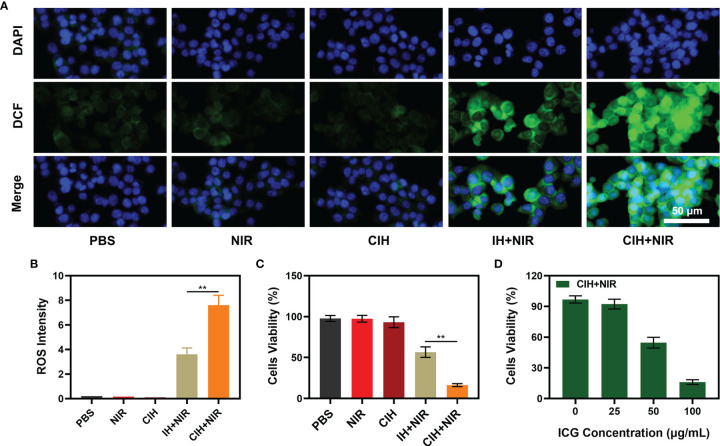
*In vitro* experiments. **(A)** Fluorescence images of 4T1 cells stained by DCFH-DA to indicate nanoparticle-induced ROS generation. Scar bar: 50 μm. **(B)** Corresponding quantitative analysis of ROS generation in 4A. **(C)** Viability of 4T1 cells cultured in the presence of various formulations. **(D)** Cell viability of 4T1 cells following the CIH treatments under different ICG concentration. **P < 0.01; Student’s t-test.

The good antitumor effect of PDT mainly comes from the direct cytotoxic effect on tumor cells and the indirect destruction of TME to regulate cell death (38–41). A large amount of ROS is generated during the PDT process (42). Based on this, we used DCFH-DA to verify the content of ROS in cells treated with different groups. In the presence of ROS, DCFH is oxidized to form a fluorescent substance DCF, and the green fluorescence intensity is proprtional to the intracellular reactive oxygen species level (43). The control group and the NIR-treated group exhibited weak green fluorescence, which is the interference signal of endogenous H_2_O_2_ in the TME (**Figure 4A**). Although Cu-Hemin could induce a fast decline of cellular GSH contents, encapsulation of hydrogels hampered its efficiency of diffusion to tumor cells. As a result, the CIH group exhibited a completely different difference in fluorescence intensity with or without NIR treatment. The IH + NIR group produced moderately intense green fluorescence, which was benefited from the effect of the photosensitizer ICG. The convergence of CIH with NIR achieved the strongest fluorescence effect, as ICG could generate a large amount of ROS in response to laser irradiation, furthermore, Cu-Hemin could reduce intracellular GSH to protect ROS from being consumed. Corresponding quantitative analysis of ROS generation are consistent with fluorescence images (**Figure 4B**). ROS combined with high temperature could eradicate cancer cells. We used the MTT assay kit to explore the cell viability after different treatments, and the results are shown in **Figure 4C**. The efficacy of ICG-based PTT/PDT was limited by TME, while the introduction of Cu-Hemin greatly enhanced the efficacy of PDT. Cell viability between CIH + NIR and IH + NIR groups had significant difference. We fixed the concentration of Cu-Hemin to tackle with TME, and changed the concentration of ICG to perform gradient control experiments, the relative results were shown in **Figure 4D**. It is difficult to evoke cell death in the presence of low concentration of ICG + power laser, and the killing effect increases with the increase of ICG concentration.

**Figure 5:**
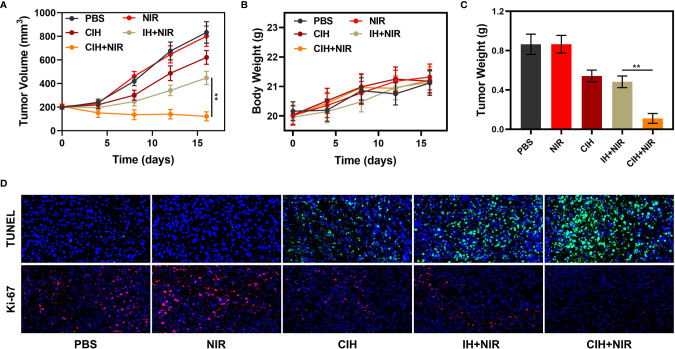
*In vivo* experiments. **(A)** Relative changes of tumor volume in mice bearing 4T1 tumors after indicated treatments. **(B)** Body weight changes of treated mice. **(C)** Tumor weight measured following the indicated treatments. **(D)** Representative digital photos of tumors collected from various groups. **(D)** TUNEL and Ki-67 tumor sections from the indicated treatment groups. **P < 0.01; Student’s t-test.

Many photothermal agents are used to achieve PTT, although many gold nanomaterials have localized surface plasmon resonance (LSPR) properties, they could strongly absorb near-infrared light and efficiently convert the light into localized heat, thereby generating heat (44, 45). However, the photothermal conversion efficiency of gold nanoparticles at 808 nm is less than 20%, which obviously cannot meet the clinical needs (13). Carbon-based materials are widely used in photothermal therapy, including graphene oxide and graphene quantum dots (46, 47). ICG has good biocompatibility and NIR light-absorbing abilities, which makes it have good clinical potential in photothermal/photodynamic therapy. We continued to explore the light-heat responsive effect of CIH *in vivo*. Compared with the control group, the tumor temperature of the mice treated by CIH + NIR group increased by about 17°C after 10 minutes, which is conducive to killing tumor cells (**Figures S3, S4**). In view of the above results, we continued to explore CIH-based *in vivo* anti-tumor effect. In this study, 4T1 cells were injected subcutaneously into the mice to establish a 4T1 breast cancer subcutaneous tumor model. As tumors reached approximately 200 mm^3^ in size, tumor-bearing BALB/c mice were randomly assigned into five groups: (1) PBS; (2) NIR; (3) CIH; (4) IH + NIR and (5) CIH + NIR. Each group was given corresponding treatment. As shown in the **Figure 5A**, the NIR group and the PBS group alone hardly had any tumor suppressive effect, and IH-based PTT/PDT produced a moderate tumor suppression rate. As CIH partly exchanges with external media, a small amount of Cu-Hemin penetrates into the tumor site to achieve slightly antitumor effect. Notably, CIH combined with NIR group could eliminate tumor tissues, as Cu-Hemin could impart oxidative damage toward the tumor, then, ICG fully utilized abnormal TME modulated by Cu-Hemin to intensify PDT. And our treatment was safe, as the mice body weight did not change drastically during the treatment period (**Figure 5B**), but increased slowly. After the treatment, we took vital organs (heart, liver, spleen, lungs, and kidney) of the mice for pathological analysis, and the results also showed that the mice did not have any inflammation and adverse conditions (**Figure S5**). This result is clinically significant, as many cargos permitted good therapeutic effects, but are also associated with high long-term risks. **Figure 5C** showed that the mice tumor weight and volume change curve is consistent. Furthermore, after the treatment of CIH combined with laser irradiation group, tumor proliferation signal Ki-67 was significantly inhibited (**Figure 5D** **).** Terminal deoxynucleotidyl transferase-mediated dUTP-biotin nick end labeling (TUNEL) also demonstrated CIH + NIR group induced profound cell apoptosis. Our designed GSH-consuming strategy amplified the ICG-mediated PDT efficiency, PTT also assisted certain therapeutic effects without any side effects.

## Conclusion

In conclusion, our designed CIH-based PTT/PDT can induce a large amount of ROS, which can damage cellular and protein structures, thereby inducing tumor cell apoptosis. The over-expressed GSH in the tumor microenvironment was also down-regulated by Cu-Hemin, thereby alleviating the degree of ROS neutralization and further enhancing the therapeutic effect of PDT. Both animal and cell models demonstrate the potent therapeutic effects of our combined system. Hydrogels with safe properties will benefit further clinical applications in the future, and we will continue to develop novel nano-systems for biological applications.

## Data Availability Statement

The original contributions presented in the study are included in the article/**Supplementary Material**. Further inquiries can be directed to the corresponding author.

## Ethics Statement

The animal experiments were carried out according to the protocol approved by the Ministry of Health in People’s Republic of PR China and were approved by Wuhan University.

## Author Contributions

SZ and SW: Methodology, Validation, Formal analysis, Roles, Writing – original draft. CL: Conceptualization, Writing – review and editing. ML: Conceptualization, Writing – review and editing. QH: Project administration, Funding acquisition, Formal analysis, Data curation. All authors contributed to the article and approved the submitted version.

## Funding

This work was supported by National Natural Science Foundation of China (31800085).

## Conflict of Interest

The authors declare that the research was conducted in the absence of any commercial or financial relationships that could be construed as a potential conflict of interest.

## Publisher’s Note

All claims expressed in this article are solely those of the authors and do not necessarily represent those of their affiliated organizations, or those of the publisher, the editors and the reviewers. Any product that may be evaluated in this article, or claim that may be made by its manufacturer, is not guaranteed or endorsed by the publisher.
