# Supplementary material for: Cu-Hemin Nanosheets and Indocyanine Green Co-Loaded Hydrogel for Photothermal Therapy and Amplified Photodynamic Therapy
Source: Front Oncol. 2022 Jun 30;12:918416. doi: 10.3389/fonc.2022.918416 (PMC9280130; doi:10.3389/fonc.2022.918416)
Supplement: Supplementary file 1 [file DataSheet_1.docx]

**Experimental Procedures**

**Materials and reagents.**

Indocyanine green (ICG), Chloro(protoporphyrinato)iron (III) (Hemin), copper(Ⅱ) nitrate trihydrate (Cu (NO3)2·3H2O) and polyvinyl pyrrolidone (PVP) were obtained from J&K Scientific Ltd. Agarose was purchased from Yare Shanghai. Reactive Oxygen Species Assay Kit, and MTT Cell Proliferation Assay Kit were obtained from Yeasen Biotech Co., Ltd (China). The other reagents used in this work were purchased from Sinopharm Chemical Reagent (China) and Aladdin-Reagent (China).

**Synthesis of Cu-Hemin nanosheets**

In brief, hemin (0.0245 mmol) was suspended in 2 mL of DMF and added into 98 mL PBS (pH value 7). Cu (NO3)2·3H2O (483 mg) and PVP (100 mg) were suspended into 100 mL distilled water and confused with the hemin solution. After stirring for 12 h, the black products were obtained by centrifugal sedimentation and cleaned with DMF and ethanol.

**Samples characterization**

Transmission electron microscopy (TEM, LIBRA 200 CS, Carl Zeiss Co., Germany) was measured to characterize the morphology of Cu-Hemin nanosheets. XPS (X-ray photoelectron spectroscopy) was constructed to analysis the elements and the chemical states of Cu-Hemin. XRD (X-ray diffraction, Empyrean, PANalytical B.V., Holland)

was employed to analysis the Cu-Hemin. Zeta potential measurements (Nano ZS90 Zetasizer, Malvern Instruments Co. Ltd, UK) were employed to verify the stability of Cu-Hemin nanosheets.

**Consumption of GSH**

Briefly, 10 mM GSH aqueous solution was mixed with different concentrations of Cu-Hemin nanosheets. After reacting for 24 h, the concentration of GSH was detected by a Reduced Glutathione (GSH) Assay Kit (BC1175, Solarbio). All experimental groups were normalized with the control group (n=3).

**Cell culture**

4T1 mouse breast cancer cell line was obtained from the Cell Bank of the Chinese Academy of Sciences and incubated in RPMI-1640 medium supplemented with 10% FBS in a humidified atmosphere at 37℃.

**Preparation and characterization of Cu-Hemin and ICG co-loaded hydrogel (CIH)**

The general protocol for the hydrogel preparation is as follows. The prepared Cu-Hemin (1 mg/mL in PBS) and the ICG (2 mg/mL in PBS) were mixed into 1% agarose solution to form CIH. Wherein the concentration of Cu-Hemin and ICG was 20 and 100 μg/mL, respectively. Photothermal heating curves of CIH at different concentrations (ICG: 0, 20 and 100 μg/mL) under an 808 nm laser irradiation was monitored using an infrared camera (Fotric 225).

**Rheological Test**

Rheology experiments were performed on an Anton Paar rheometer. Hydrogel samples of different temperatures were prepared and gently placed on the middle of a 15 mm diameter parallel plate with a proper gap. Dynamic oscillatory frequency sweep measurements were conducted at a 1% strain amplitude. To prevent the evaporation of water, a lid was prepared on the top.

**Intracellular reactive oxygen species (ROS) generation**

For determination of ROS levels via fluorescent imaging, 4T1 cells were incubated for 2 h with 5 different groups: (1) PBS, (2) NIR (0.5 W/cm^2^, 5min) (3) CIH (4) IH+NIR (5) CIH+NIR. The ICG concentration was 100 μg/mL in group 3, 4 and 5. Then, the fluorescent dye, DCFH-DA (10 μmol/L), was added and co-incubated for 20 min at 37 °C. Then, cells in group 2, 4 and 5 were irradiated with the NIR. ROS level was determined by a confocal laser scanning microscope (CLSM; IX81, Olympus, Japan). The fluorescent intensity of each group was calculated by ImageJ software.

***In vitro* anti-tumor ability of CIH**

4T1 cells were seeded in 96-well plates at a density of 5 × 10^3^ cells per well and incubated for 24 h. Afterwards, 4T1 cells were incubated for 5 different groups: (1) PBS, (2) NIR (0.5 W/cm^2^, 5min) (3) CIH (4) IH+NIR (5) CIH+NIR. The ICG concentration was 100 μg/mL in group 3, 4 and 5. Then, Then, cells in group 2, 4 and 5 were irradiated with the NIR. At the end of the incubation, 5 mg/mL MTT PBS solution was added, and the plate was incubated for another 4 h. Finally, the absorbance values of the cells were determined by using a microplate reader (Emax Precision, USA) at 570 nm. The background absorbance of the well plate was measured and subtracted. The cytotoxicity was calculated by dividing the optical density (OD) values of treated groups (T) by the OD values of the control (C) (T/C × 100%). The anti-tumor ability of CIH with different concentration were conducted as the same method.

**Animal tumor models**

Female BALB/c mice aged 4-5 week were purchased from Vital River Company (Beijing, China). 100 μL of 4T1 cell suspension (1×10^6^ cells per mL) were subcutaneous injected into each mouse to establish the tumor models. The animal experiments were carried out according to the protocol approved by the Ministry of Health in People’s Republic of PR China and were approved by the Administrative Committee on Animal Research of the second clinical Medicine College of Wuhan University.

***In vivo* infrared thermography**

To monitor the *in vivo* photothermal effect, CIH (Cu-hemin: 2mg/kg, ICG: 10 mg/kg) was intratumorally injected into the tumor-bearing mice, and then the tumors suffered from 0.5 W/cm^2^ irradiation for 10 min at 0.5 h post-injection. PBS injection used as control group. Meanwhile, the temperature at the tumor was monitored using an infrared camera (Fotric 225).

***In vivo* antitumor study**

After the tumor size reached 200 mm^3^, the mice were divided randomly into 5 groups (n =3 per group): (1) PBS, (2) NIR (0.5 W/cm^2^, 10min) (3) CIH (4) IH + NIR (5) CIH + NIR. Among them, the dose of Cu-Hemin in groups 3 and 5 are 2 mg/kg. The dose of ICG in groups 3, 4 and 5 are 10 mg/kg. The injection method is intratumoral injection. After 0.5h injection, the tumor site of mice in groups 2, 4 and 5 were treated by NIR. Mice body weight and tumor volume in all groups were monitored every 4 days. A caliper was employed to measure the tumor length and tumor width and the tumor volume was calculated according to following formula. Tumor volume = tumor length × tumor width^2^ / 2. After 16 days treatment, mice were sacrificed. Five main organs (heart, liver, spleen, lung and kidney) of all mice were harvested, washed with PBS, and fixed with paraformaldehyde for histology analysis. The blood samples from these mice (≈1 mL) were collected for blood biochemistry analysis. And the tumor tissues were weighed, and fixed in 4% neutral buffered formalin, processed routinely into paraffin, and sectioned at 4 μm. Then the sections were stained with TUNEL and Ki-67.

**Statistical analysis**

Data analyses were conducted using the GraphPad Prism 5.0 software. Significance between every two groups was calculated by the student’s t-test. *P < 0.05, **P < 0.01, ***P < 0.005.


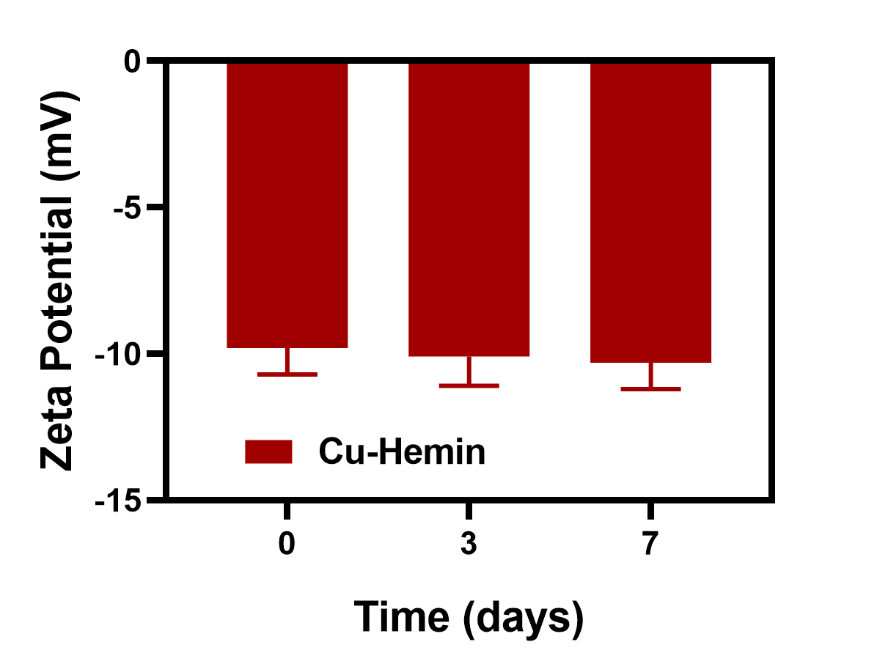


**Figure S1.** Zeta potential values for Cu-Hemin nanosheets.





**Figure S2**. SEM images of hydrogels.


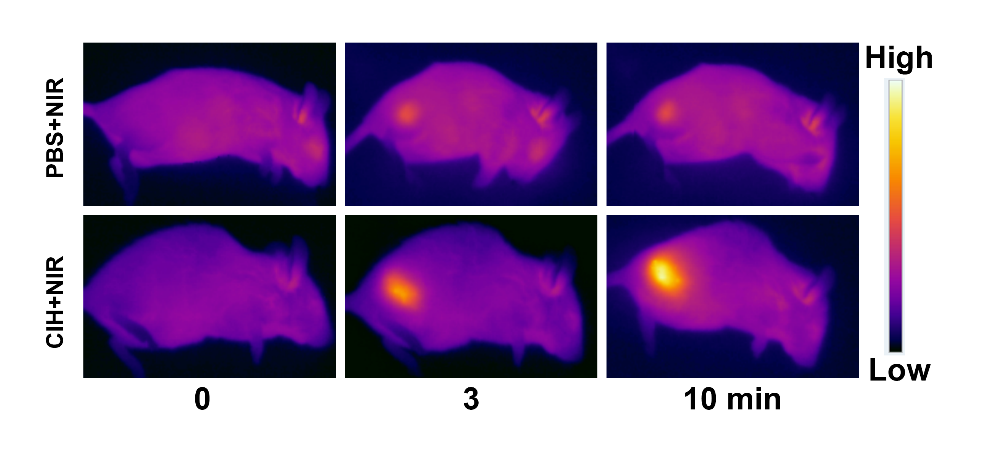


**Figure S3**. Infrared images of the mice tissue under laser irradiation after the indicated treatments.


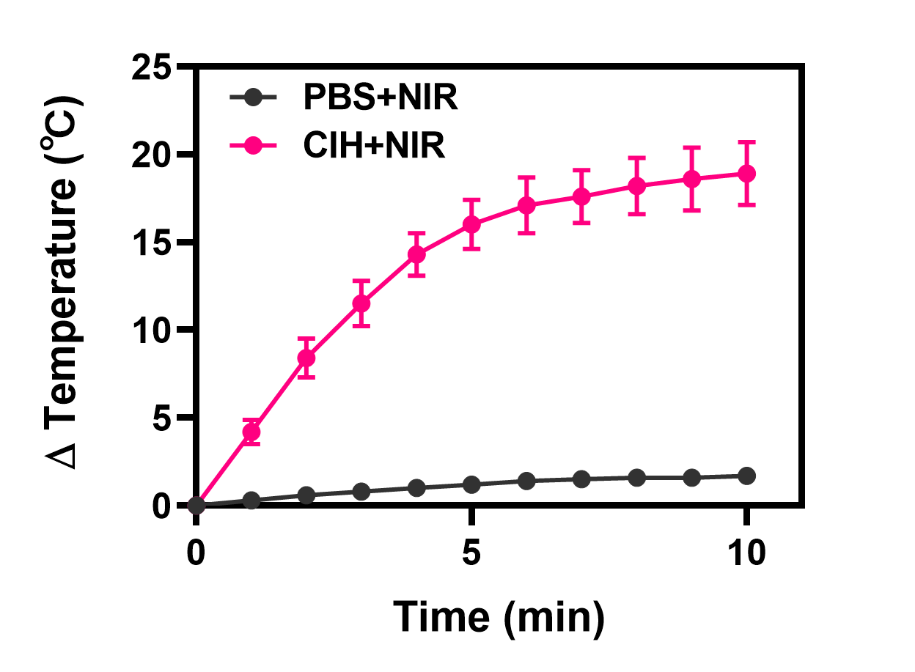


**Figure S4**. Temperature increases in mice implanted with 4T1 tumors following 808 nm laser irradiation (0.5 W/cm^2^) for 5 min in the indicated treatment groups.


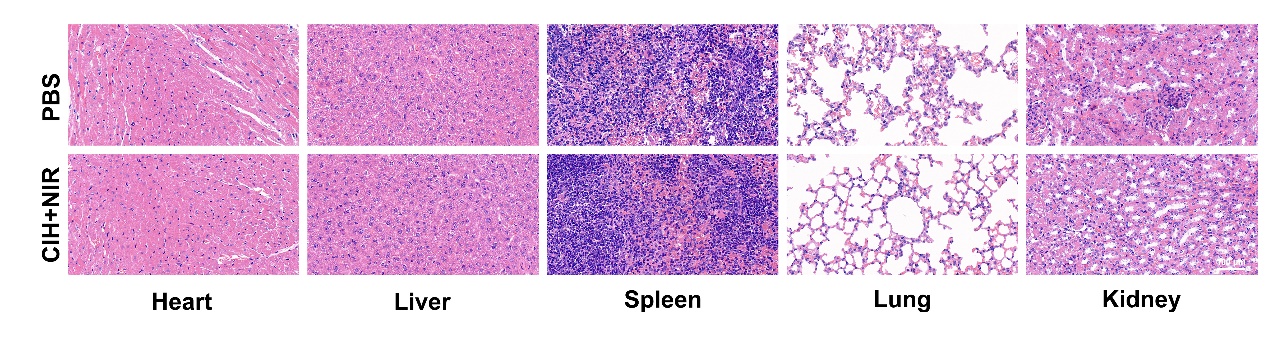


**Figure S5**. Histopathological analysis results (H&E stained images) of the major organs, heart, lung, liver, kidneys, and spleen, of mice that were exposed to different treatments 16 days post-injection.
